# Historical Redlining, Persistent Mortgage Discrimination, and Race in Breast Cancer Outcomes

**DOI:** 10.1001/jamanetworkopen.2023.56879

**Published:** 2024-02-20

**Authors:** Jasmine M. Miller-Kleinhenz, Lauren E. Barber, Maret L. Maliniak, Leah Moubadder, Maya Bliss, Micah J. Streiff, Jeffrey M. Switchenko, Kevin C. Ward, Lauren E. McCullough

**Affiliations:** 1 Department of Epidemiology, Rollins School of Public Health, Emory University, Atlanta, Georgia; 2 Department of Biostatistics, Rollins School of Public Health, Emory University, Atlanta, Georgia

## Abstract

**Importance:**

Inequities created by historical and contemporary mortgage discriminatory policies have implications for health disparities. The role of persistent mortgage discrimination (PMD) in breast cancer (BC) outcomes has not been studied.

**Objective:**

To estimate the race-specific association of historical redlining (HRL) with the development of BC subtypes and late-stage disease and a novel measure of PMD in BC mortality.

**Design, Setting, and Participants:**

This population-based cohort study used Georgia Cancer Registry data. A total of 1764 non-Hispanic Black and White women with a BC diagnosis and residing in an area graded by the Home Owners’ Loan Corporation (HOLC) in Georgia were included. Patients were excluded if they did not have a known subtype or a derived American Joint Committee on Cancer stage or if diagnosed solely by death certificate or autopsy. Participants were diagnosed with a first primary BC between January 1, 2010, to December 31, 2017, and were followed through December 31, 2019. Data were analyzed between May 1, 2022, and August 31, 2023.

**Exposures:**

Scores for HRL were examined dichotomously as less than 2.5 (ie, nonredlined) vs 2.5 or greater (ie, redlined). Contemporary mortgage discrimination (CMD) scores were calculated, and PMD index was created using the combination of HRL and CMD scores.

**Main Outcomes and Measures:**

Estrogen receptor (ER) status, late stage at diagnosis, and BC-specific death.

**Results:**

This study included 1764 women diagnosed with BC within census tracts that were HOLC graded in Georgia. Of these, 856 women (48.5%) were non-Hispanic Black and 908 (51.5%) were non-Hispanic White; 1148 (65.1%) were diagnosed at 55 years or older; 538 (30.5%) resided in tracts with HRL scores less than 2.5; and 1226 (69.5%) resided in tracts with HRL scores 2.5 or greater. Living in HRL areas with HRL scores 2.5 or greater was associated with a 62% increased odds of ER-negative BC among non-Hispanic Black women (odds ratio [OR], 1.62 [95% CI, 1.01-2.60]), a 97% increased odds of late-stage diagnosis among non-Hispanic White women (OR, 1.97 [95% CI, 1.15-3.36]), and a 60% increase in BC mortality overall (hazard ratio, 1.60 [95% CI, 1.17-2.18]). Similarly, PMD was associated with BC mortality among non-Hispanic White women but not among non-Hispanic Black women.

**Conclusions and Relevance:**

The findings of this cohort study suggest that historical racist policies and persistent discrimination have modern-day implications for BC outcomes that differ by race. These findings emphasize the need for a more nuanced investigation of the social and structural drivers of disparate BC outcomes.

## Key Points

**Question:**

Are historical and persistent mortgage discrimination associated with current breast cancer outcomes?

**Findings:**

In a cohort study of 1764 women with breast cancer, living in a historically redlined area was associated with increased odds of a diagnosis of estrogen receptor–negative breast cancer in non-Hispanic Black women and increased odds of late-stage diagnosis in non-Hispanic White women. Persistent mortgage discrimination was associated with an increase in breast cancer mortality in non-Hispanic White women, and non-Hispanic Black women were more likely to die of breast cancer no matter where they lived.

**Meaning:**

These findings suggest that historical and persistent mortgage discrimination have modern-day implications for breast cancer outcomes.

## Teaser

This cohort study assesses the association of neighborhood-level historical redlining with estrogen receptor status and stage at diagnosis of breast cancer and the association of contemporary and persistent mortgage discrimination with breast cancer mortality among non-Hispanic Black and White women in metropolitan areas of Georgia.

## Introduction

Breast cancer (BC) is one of the leading causes of cancer-related death for women in the US.^1^ While Black women have a slightly lower incidence rate of BC than White women, they are more frequently diagnosed with advanced disease and have a higher incidence of aggressive forms of BC, such as estrogen receptor (ER)–negative disease.^2^ These differences in BC stage at diagnosis and ER status contribute to racial disparities in BC outcomes. However, there is limited understanding of how societal structures drive inequitable outcomes.^3,4^

Discriminatory policies and laws in the US have perpetuated institutional and structural racism, which have profoundly affected various aspects of society, including education, health care, economics, and housing, ultimately affecting neighborhoods.^5,6,7^ Recent research has shown that historical redlining (HRL), a form of mortgage discrimination that was largely based on the racial and ethnic composition of neighborhoods, is a contributing factor to racial disparities in BC outcomes, including diagnosis at later stages and with ER-negative BC.^8^ While the institutionalization of HRL through Home Owners’ Loan Corporation (HOLC) maps began in the 1930s and was outlawed in the 1960s by the Fair Housing Act, the practice of contemporary mortgage discrimination (CMD) persists.^9^ Historical redlining and CMD could have downstream implications for residents’ exposure to their built, socioeconomic, and chemical environments, further exacerbating racial disparities in BC outcomes.^10,11^

Although studies have examined the effect of HRL and CMD on BC outcomes separately, to our knowledge, no study has investigated the role of persistent mortgage discrimination (PMD), where neighborhoods have experienced both HRL and CMD. Our objective was to assess the association of neighborhood-level HRL with ER-status and BC stage at diagnosis and the association of CMD and PMD with BC mortality in metropolitan areas of Georgia, a state with a diverse population and pronounced racial disparities in BC outcomes.^12,13,14^

## Methods

### Study Population

In this cohort study using the Georgia Cancer Registry (GCR), we identified non-Hispanic Black and non-Hispanic White adult women who had been diagnosed with a first invasive primary stage I to IV BC (*International Classification of Diseases for Oncology, Third Edition* code C50) between January 1, 2010, to December 31, 2017, and were followed up through December 31, 2019. Data were analyzed between May 1, 2022, and August 31, 2023.

Women were included if they resided in an HOLC-graded census tract (n = 1703) in Georgia at the time of diagnosis, including the metropolitan areas of Atlanta, Augusta, Columbus, Macon, and Savannah. Patients were excluded if they did not have a known molecular subtype or a derived American Joint Committee on Cancer stage or if diagnosed solely by death certificate or autopsy findings. The GCR captures the address at the time of diagnosis for each patient with cancer and geocodes all addresses to the census tract level. This study followed the Strengthening the Reporting of Observational Studies in Epidemiology (STROBE) reporting guideline. The institutional review board of Emory University approved this study, and a waiver of informed consent was granted given the nature of the study and the use of limited personal identifiable GCR data.

### Exposure Assessment

#### Historical Redlining

The HRL score was defined according to the digitized maps provided by the Mapping Inequality Project at the University of Richmond.^15^ Historical redlining was calculated using the weighted score method as previously described by Meier and Mitchell^16^ (eMethods in Supplement 1). Historical redlining scores were examined dichotomously: less than 2.5 represents areas that were historically considered low risk for investment (grade A [green] indicated “best”; grade B [blue], “still desirable” [ie, nonredlined]), and 2.5 or greater represents areas that were considered to be high risk (grade C [yellow] indicated “definitely declining”; grade D [red], “hazardous” [ie, redlined]).

#### Contemporary Mortgage Discrimination

To measure CMD based on location, we calculated a CMD score. This score was calculated based on a published methodologic approach by Mendez et al^17^ and Beyer et al^18^ (eMethods in Supplement 1). A value less than 1 indicated that applicants in the tract were less likely to be denied mortgage applications, whereas a value of 1 or greater indicated that applicants in the tract were more likely to be denied mortgage applications than applicants in other tracts. The odds ratios (ORs) for CMD were calculated using logistic regression at the tract level using data from the Home Mortgage Disclosure Act, adjusting for applicant sex and ratio of loan to income. We then assigned CMD scores according to the patient’s census tract at the time of their BC diagnosis.

#### Persistent Mortgage Discrimination

We created a measure of PMD using the combination of HRL and CMD scores. We classified tracts as experiencing PMD if they were historically redlined (HRL score ≥2.5) and continued to experience CMD (CMD score ≥1) (625 tracts). Other combination groups included HRL neighborhoods that did not experience CMD (HRL score ≥2.5 and CMD score <1) (533 tracts), non-HRL neighborhoods that experienced CMD (HRL score <2.5 and CMD score ≥1) (231 tracts), and non-HRL neighborhoods that did not experience CMD (HRL score <2.5 and CMD score <1) (314 tracts). The latter group served as the reference population. Historical redlining, CMD, and PMD are graphically represented for each city within the study in the Figure and in eFigure 1 in Supplement 1.

**Figure.**
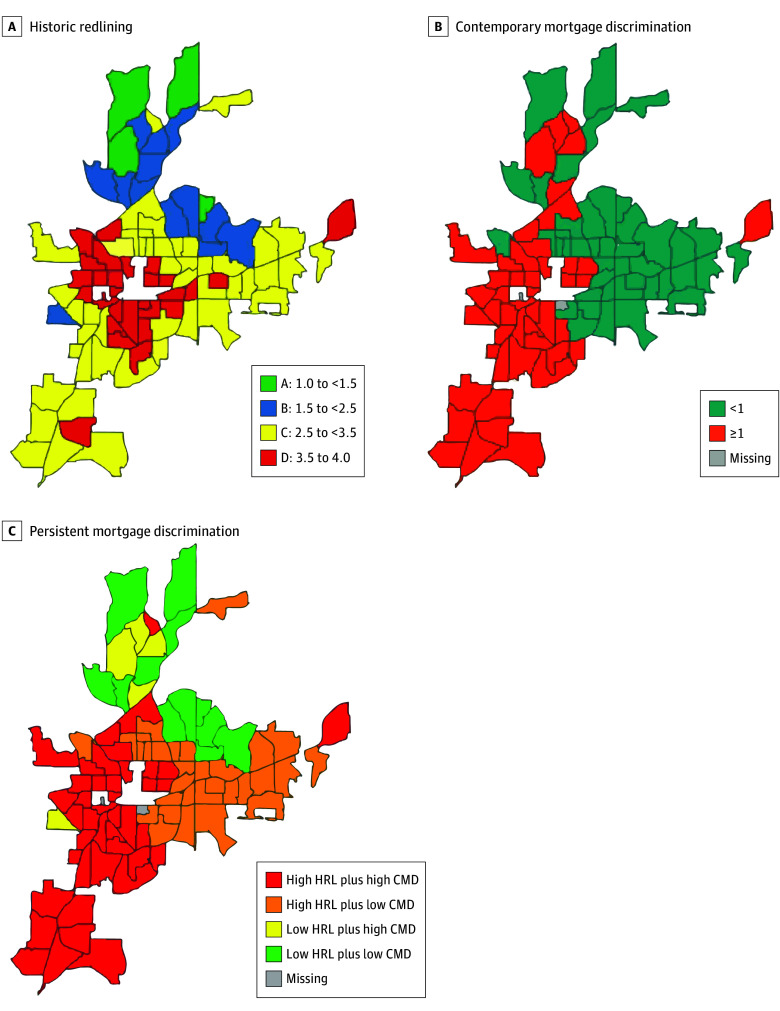
Distribution of Historical Redlining (HRL), Contemporary Mortgage Discrimination (CMD), and Persistent Mortgage Discrimination (PMD) in Atlanta, 2010 to 2017 A, HRL is mapped by Home Owners’ Loan Corporation (HOLC) grade. Grade A (green) indicates “best”; grade B (blue), “still desirable”; grade C (yellow), “definitely declining”; and grade D (red), “hazardous.” HRL scores less than 2.5 indicate grade A or B (nonredlined); scores of 2.5 or greater, grade C or D (redlined). B, CMD scores are mapped as less than 1 (applicants in the tract are less likely to be denied mortgage applications) vs 1 or greater (applicants in the tract were more likely to be denied mortgage applications). C, PMD was calculated as a combination of HRL and CMD scores.

### Outcome Assessment

Estrogen receptor status was based on the expression of ER and was used to dichotomize patients into ER-positive and ER-negative groups. Derived American Joint Committee on Cancer stage was used to dichotomize patients as having early (stage I-IIIa) and late (stage IIIb-IV) BC.

Underlying cause of death was determined directly from death certificates using *International Statistical Classification of Diseases and Related Health Problems, Tenth Revision* codes. In this study, we included BC-related deaths (code C50) recorded through December 31, 2019, as events in the survival models.

### Covariates of Interest

#### Patient Characteristics

Race and ethnicity were obtained by the GCR using medical records and other information from the reporting facility.^19^ Age at diagnosis, marital status (eg, married [common law and unmarried domestic] or other [divorced, widowed, or separated]), and insurance at diagnosis (private, Medicare, Medicaid, uninsured, or other) were obtained from the GCR records.

#### Neighborhood Characteristics

Neighborhood characteristics were derived using data from the American Community Survey and calculated at the census tract level using 5-year estimates from 2010 to 2014.^20^ Characteristics included proportion of the population identified as Black (≥50% or <50%), percentage of the population living below the federal poverty level (≥20% or <20%), percentage of the population 25 years and older without a high school diploma (≥20% or <20%), and median household income.

### Statistical Analysis

Descriptive statistics were calculated for covariates across categorizations of HRL scores (<2.5 vs ≥2.5) as frequencies and proportions. In race-stratified models, we used age-adjusted logistic regression to calculate the ORs and 95% CIs associating HRL with ER status (negative vs positive) and stage (late vs early). We used age-adjusted and multivariable-adjusted Cox proportional hazards regression models to calculate the hazard ratios (HRs) and 95% CIs for the associations of BC mortality with HRL and PMD. The accrual of person-time started at the time of diagnosis. Potential confounders were determined a priori based on previous literature and graphical-based methods.^21^ Our graphical assessment of potential confounders showed that all covariates of interest were on the causal path between redlining, ER status, stage, and BC mortality (eFigure 2 in Supplement 1). Thus, we focus on age-adjusted results. Analyses were performed using SAS, version 9.4 (SAS Institute Inc).

## Results

We identified 1764 women diagnosed with BC within census tracts that were HOLC graded in Georgia for our study. Among these women, 856 (48.5%) were non-Hispanic Black and 908 (51.5%) were non-Hispanic White; 1148 (65.1%) were diagnosed at 55 years or older; 538 (30.5%) resided in tracts with HRL scores less than 2.5; and 1226 (69.5%) resided in tracts with HRL scores 2.5 or greater (Table 1). Patients with BC residing in areas with HRL scores 2.5 or greater were more likely to have ER-negative disease (271 of 1226 [22.1%] vs 75 of 538 [13.9%]) and late-stage disease at diagnosis (178 of 1226 [14.5%] vs 48 of 538 [8.9%]) compared with women residing in areas with HRL scores less than 2.5 (Table 1).

**Table 1.** Patient Demographic, Clinicopathological, and Treatment Characteristics^a^

Characteristic	Patient cohort, No. (%)^b^		
	Overall (N = 1764)	HRL score^c^	
		<2.5 (n = 538)	≥2.5 (n = 1226)
Race and ethnicity			
Non-Hispanic Black	856 (48.5)	119 (22.1)	737 (60.1)
Non-Hispanic White	908 (51.5)	419 (77.9)	489 (39.9)
ER status			
Positive	1418 (80.4)	463 (86.1)	955 (77.9)
Negative	346 (19.6)	75 (13.9)	271 (22.1)
Disease stage			
Early	1538 (87.2)	490 (91.1)	1048 (85.5)
Late	226 (12.8)	48 (8.9)	178 (14.5)
Age at diagnosis, y			
<55	616 (34.9)	166 (30.9)	450 (36.7)
≥55	1148 (65.1)	372 (69.1)	776 (63.3)
Marital status			
Single	467 (26.5)	101 (18.8)	366 (29.9)
Married (common law and unmarried domestic)	660 (37.4)	286 (53.2)	374 (30.5)
Other (divorced, widowed, or separated)	530 (30.1)	133 (24.7)	397 (32.4)
No. unknown	107	18	89
Insurance type			
Private	872 (49.4)	309 (57.4)	563 (45.9)
Medicare	593 (33.6)	176 (32.7)	417 (34.0)
Medicaid	213 (12.1)	31 (5.8)	182 (14.8)
Uninsured	40 (2.3)	9 (1.7)	31 (2.5)
Other	24 (1.4)	6 (1.1)	18 (1.5)
No. unknown	22	7	15
Breast cancer–specific death	228 (12.9)	52 (9.7)	176 (14.4)
Neighborhood			
Proportion of Black residents			
≥50%	707 (40.1)	99 (18.4)	608 (49.6)
<50%	1057 (59.9)	439 (81.6)	618 (50.4)
Proportion living below federal poverty line			
≥20%	895 (50.7)	115 (21.4)	780 (63.6)
<20%	869 (49.3)	423 (78.6)	446 (36.4)
Proportion with less than high school education			
≥20%	352 (20.0)	24 (4.5)	328 (26.8)
<20%	1412 (80.0)	514 (95.5)	898 (73.2)
Median household income, $			
<44 311	877 (49.7)	122 (22.7)	755 (61.6)
44 311 to <61 403	198 (11.2)	56 (10.4)	142 (11.6)
61 403 to <84 497	275 (15.6)	88 (16.4)	187 (15.3)
≥84 497	414 (23.5)	272 (50.6)	142 (11.6)

Abbreviations: ER, estrogen receptor; HRL, historical redlining.

^a^ Participants were diagnosed with stages I to IV breast cancer in Georgia and included in the Georgia Cancer Registry between 2010 and 2017.

^b^ Percentages have been rounded and may not total 100.

^c^ Scores less than 2.5 indicate no redlining; scores 2.5 or greater indicate redlining.

### HRL and BC ER Status and Stage

In the age-adjusted model, women who lived in areas with HRL scores 2.5 or greater had 1.72-fold increased odds of diagnosis with ER-negative BC compared with those who did not (95% CI, 1.30-2.27) (Table 2). The association between HRL and ER status differed by race (Table 2). Non-Hispanic Black women who resided in areas with HRL scores 2.5 or greater had a 1.62-fold increase in the odds of diagnosis with ER-negative BC (95% CI, 1.01-2.60) compared with those living in areas with HRL scores less than 2.5. Among non-Hispanic White women residing in areas with HRL scores 2.5 or greater, we observed a decrease in the odds of diagnosis with ER-negative BC (OR, 0.85 [95% CI, 0.57-1.28]). When we examined these associations by BC subtype, we observed a 1.32-fold increase in the odds of diagnosis with triple-negative BC (95% CI, 0.80-2.16) among non-Hispanic Black women and a slight increase among non-Hispanic White women (OR, 1.08 [95% CI, 0.72-1.62]) (eTable 1 in Supplement 1).

**Table 2.** ER Status and Cancer Stage According to Census Tract HRL Score^a^

HRL score^b^	ER status, No. of women		Age-adjusted OR (95% CI)	Stage, No. of women		Age-adjusted OR (95% CI)
	Positive	Negative		Early	Late	
**All participants**						
Continuous	1418	346	1.44 (1.24-1.68)	1538	226	1.56 (1.29-1.87)
Dichotomous						
<2.5	463	75	1 [Reference]	490	48	1 [Reference]
≥2.5	955	271	1.72 (1.30-2.27)	1048	178	1.75 (1.25-2.45)
**Non-Hispanic Black women**						
Continuous	616	240	1.20 (0.96-1.50)	696	160	0.93 (0.72-1.20)
Dichotomous						
<2.5	95	24	1 [Reference]	93	26	1 [Reference]
≥2.5	521	216	1.62 (1.01-2.60)	603	134	0.78 (0.49-1.26)
**Non-Hispanic White women**						
Continuous	802	106	0.93 (0.71-1.21)	842	66	1.88 (1.33-2.66)
Dichotomous						
<2.5	368	51	1 [Reference]	397	22	1 [Reference]
≥2.5	434	55	0.85 (0.57-1.28)	445	44	1.97 (1.15-3.36)

Abbreviations: ER, estrogen receptor; HRL, historical redlining; OR, odds ratio.

^a^ Participants were diagnosed with stages I to IV breast cancer in Georgia and included in the Georgia Cancer Registry between 2010 and 2017.

^b^ Scores less than 2.5 indicate no redlining; scores 2.5 or greater indicate redlining.

In the age-adjusted model, women who lived in areas with HRL scores 2.5 or greater had 1.75-fold increased odds of late-stage diagnosis compared with those who did not (95% CI, 1.25-2.45) (Table 2). We similarly observed differences by race for the association between HRL score and late stage at diagnosis (Table 2). Non-Hispanic Black women residing in areas with HRL scores 2.5 or greater had a lower odds of late-stage diagnosis compared with non-Hispanic Black women who lived in areas with HRL scores less than 2.5 (OR, 0.78 [95% CI, 0.49-1.26]), whereas non-Hispanic White women who resided in areas of HRL scores 2.5 or greater were more likely to be diagnosed at a later stage compared with non-Hispanic White women residing in areas with HRL scores less than 2.5 (OR, 1.97 [95% CI, 1.15-3.36]) (Table 2).

### HRL and BC Mortality

In the age-adjusted models, women who lived in areas with HRL scores 2.5 or greater had 1.60 times the estimated rate of BC mortality compared with those who did not (95% CI, 1.17-2.18) (Table 3). This association attenuated to the null in the multivariable-adjusted model (HR, 0.96 [95% CI, 0.69-1.34]). Adjustment for competing risks of death using Fine-Gray did not change our results. We observed no association between HRL and BC mortality among non-Hispanic Black women in age- or multivariable-adjusted models (age-adjusted HR, 0.88 [95% CI, 0.58-1.33]). Among non-Hispanic White women, we observed an association of HRL score with BC mortality (HR, 1.31 [95% CI, 0.79-2.18]), which persisted in the multivariable-adjusted model (HR, 1.28 [95% CI, 0.76-2.14]). We also conducted age-adjusted cumulative incidence function analyses, which were consistent with the hazard regression analyses (eFigure 3 in Supplement 1).

**Table 3.** Hazards for Breast Cancer–Specific Mortality According to Census Tract HRL Score^a^

HRL score^b^	No. of deaths	Age-adjusted HR (95% CI)	MV-adjusted HR (95% CI)^c^	Additional MV-adjusted HR (95% CI)^d^
**All participants**				
Continuous	228	1.43 (1.20-1.69)	1.19 (1.01-1.40)	1.07 (0.90-1.28)
Dichotomous				
<2.5	52	1 [Reference]	1 [Reference]	1 [Reference]
≥2.5	176	1.60 (1.17-2.18)	1.18 (0.87-1.62)	0.96 (0.69-1.34)
**Non-Hispanic Black women**				
Continuous	166	0.94 (0.76-1.17)	1.00 (0.80-1.25)	NA
Dichotomous				
<2.5	26	1 [Reference]	1 [Reference]	NA
≥2.5	140	0.88 (0.58-1.33)	0.80 (0.56-1.22)	NA
**Non-Hispanic White women**				
Continuous	62	1.37 (0.99-1.90)	1.17 (0.87-1.57)	NA
Dichotomous				
<2.5	26	1 [Reference]	1 [Reference]	NA
≥2.5	36	1.31 (0.79-2.18)	1.28 (0.76-2.14)	NA

Abbreviations: HR, hazard ratio; HRL, historical redlining; MV, multivariable; NA, not applicable.

^a^ Participants were diagnosed with stages I to IV breast cancer in Georgia and included in the Georgia Cancer Registry between 2010 and 2017.

^b^ Scores less than 2.5 indicate no redlining; scores 2.5 or greater indicate redlining.

^c^ Adjusted for age, stage, and estrogen receptor status.

^d^ Adjusted for age, stage, estrogen receptor status, and race.

### PMD and BC ER Status, Stage, and Mortality

We assessed the association between PMD and ER status, stage at diagnosis, and BC mortality, comparing each race and exposure combination with non-Hispanic White women who were doubly unexposed (HRL score <2.5 and CMD <1). Among non-Hispanic White women, those exposed to PMD had a modest association for odds of a diagnosis of ER-negative BC (OR, 1.19 [95% CI, 0.66-2.14]) and a more than 3-fold odds of late-stage diagnosis (OR, 3.74 [95% CI, 1.90-7.38]) (eTable 2 in Supplement 1). Among non-Hispanic Black women, we observed elevated odds of ER-negative BC and late-stage diagnosis compared to non-Hispanic White women across combinations of HRL and CMD (eTable 2 in Supplement 1).

Among non-Hispanic White women, those exposed to PMD were twice as likely to die of BC compared with their non-Hispanic White counterparts residing in areas without HRL or CMD (HR, 2.17 [95% CI, 1.13- 4.18]). Other combinations of HRL and CMD scores had associations that were modest or near null (Table 4). Among non-Hispanic Black women, we observed a more than 3-fold elevated hazard of BC mortality in age-adjusted models irrespective of the combination of HRL or CMD, although we did not have enough data to compare the groups with low HRL and low CMD (Table 4).

**Table 4.** Hazards for Breast Cancer–Specific Mortality According to Census Tract HRL and CMD Indices^a^

Race by PMD	No. of deaths	HR (95% CI)	
		Age-adjusted	MV-adjusted^b^
Non-Hispanic White women			
Low HRL plus low CMD	18	1 [Reference]	1 [Reference]
High HRL plus low CMD	18	0.88 (0.46-1.69)	1.05 (0.54-2.02)
Low HRL plus high CMD	8	1.02 (0.44-2.35)	1.06 (0.46-2.45)
High HRL plus high CMD	18	2.17 (1.13-4.18)	1.45 (0.75-2.80)
Non-Hispanic Black women^c^			
High HRL plus low CMD	32	3.23 (1.81-5.76)	1.78 (0.99-3.19)
Low HRL plus high CMD	23	4.02 (2.17-7.46)	2.28 (1.22-4.25)
High HRL plus high CMD	94	3.74 (2.26-6.20)	1.99 (1.19-3.31)

Abbreviations: CMD, contemporary mortgage discrimination; HR, hazard ratio; HRL, historical redlining; MV, multivariable; PMD, persistent mortgage discrimination.

^a^ Participants were diagnosed with stages I to IV breast cancer in Georgia and included in the Georgia Cancer Registry between 2010 and 2017.

^b^ Adjusted for age, stage, and estrogen receptor status.

^c^ The group with low HRL plus low CMD is excluded due to fewer than 5 patients who died.

## Discussion

This cohort study reinforces the distinct associations that HRL and CMD have with BC outcomes, particularly by race. We found that areas with HRL had increased rates of ER-negative BC diagnoses among non-Hispanic Black women, late-stage diagnoses among non-Hispanic White women, and BC mortality among non-Hispanic White women. Persistent mortgage discrimination was associated with greater BC mortality among non-Hispanic White women, while non-Hispanic Black women had an increased hazard of BC mortality across combinations of HRL and CMD.

Race-specific differences in the development of ER-negative BC and late stage at diagnosis are likely related to different processes along the cancer continuum; the former reflects biological changes (eg, tumor initiation, promotion, and progression) and the latter is associated with access to care. Previous studies have emphasized the need to include historical and societal contexts to better understand the higher prevalence of ER-negative disease among Black women, hypothesizing that racially discriminatory practices could have long-term consequences for contemporary health outcomes.^22^ This hypothesis was nicely displayed in a study that showed that being born in a state that practiced racially discriminatory Jim Crow laws was associated with increased odds of ER-negative BC.^23^ Importantly, this effect was only observed among Black women, highlighting the impact of racially discriminatory laws on BC development. Our race-stratified results complement these findings, suggesting that disadvantage goes beyond health care access, with the potential for racist policies or practices to permeate the skin and manifest biologically.

Unlike the biological drivers that manifest in tumor subtypes, stage at diagnosis is likely a reflection of differential health care access and screening quality. Previous studies have found that living in an HRL neighborhood increases the risk of late-stage diagnoses of various types of cancer, including BC.^8,24^ Only 1 study^8^ has looked at race-specific estimates of HRL and BC outcomes and found that living in the best HOLC-graded areas was associated with lower odds of late-stage diagnosis, but only for non-Hispanic White women. Our results are consistent with findings of these studies and suggest that the stage at diagnosis for non-Hispanic White women might be driven by access to and quality of care. Non-Hispanic Black women face additional barriers (eg, systemic racism, clinician biases) that may affect their ability to receive timely, quality care irrespective of their residence.^25^

Our finding of a positive association between HRL and BC mortality is consistent with the findings of other studies that examined the effect of HRL on cancer mortality.^26,27^ While previous studies did not stratify by race, our results suggest that race is a factor in the posited association. Consistent with this, we observed that the association between HRL and BC mortality was limited to non-Hispanic White women. Our overall findings may inform areal-level interventions to reduce mortality among all women but would not inform strategies to narrow and ultimately close the BC mortality gap.

Studies that examine the effects of HRL or CMD on BC outcomes continue to emerge, but to our knowledge, none have explored the effects of persistent discrimination.^8,24,26,27,28^ Our findings suggest that non-Hispanic White women living in areas with sustained mortgage discrimination face twice the risk of BC mortality compared with non-Hispanic White women living in areas without PMD. Areas with PMD tend to have other factors contributing to poor health outcomes, such as inadequate housing, education, physical work environments, health care, nutrition, green space, physical activity, and financial stress.^29,30,31,32,33^ We also found that non-Hispanic Black women residing in a neighborhood with any combination of HRL or CMD have a greater than 3-fold excess risk of BC mortality compared with non-Hispanic White women living in areas without PMD. It is noteworthy that of the 856 non-Hispanic Black women in our study, only 15 lived in areas that did not experience HRL or CMD, limiting our assessment of outcomes in this group. These findings suggest that discrimination based on neighborhood might not be the primary cause of BC mortality disparities between non-Hispanic Black and non-Hispanic White women. It is crucial to identify various forms of discrimination and racism that disproportionately affect non-Hispanic Black women and extend beyond the neighborhood environment to understand how these factors contribute to increased risk of BC mortality.

### Strengths and Limitations

This study has several strengths. Notably, it examines the association of PMD with BC mortality. It also builds on previous research of disparities in BC survival in metropolitan Atlanta^12,13,14^—a region with a large and racially and socioeconomically diverse population—and expands to include other Georgia metropolitan areas. Additionally, this study measures the association of a historically racist policy with ER status, disease stage at diagnosis, and BC mortality in non-Hispanic Black and non-Hispanic White patients.

This study also has limitations to consider. We were unable to assess residential history or patient mobility; therefore, we assigned both HRL and CMD based on a woman’s census tract at diagnosis. Understanding the effect of neighborhood on health outcomes could be enhanced by examining neighborhood-level exposures over the life course, particularly with respect to the development of aggressive disease. Importantly, this study was limited by its small sample size. We observed noncollapsibility in our overall HRL and ER status model, though it is unclear whether this is due to the noncollapsibility of the OR, confounding, or both.^34^ Although Georgia includes approximately 11 million residents, approximately 3% reside in HRL areas, and an even smaller fraction are diagnosed with BC. While this hindered our ability to assess exposure more granularly (eg, HOLC grades D, C, and B vs A), we observed novel associations between PMD and BC mortality, and the methodology can be easily transported to other HRL areas throughout the US.

## Conclusions

In this cohort study, we investigated the association of mortgage discrimination with BC outcomes. Our results show that non-Hispanic Black women had increased odds of diagnosis with ER-negative BC when residing in HRL areas, non-Hispanic White women had increased odds of diagnosis with late-stage BC when residing in HRL areas, and non-Hispanic Black women were more likely to die of BC regardless of whether they resided in an area with HRL or PMD. Our data are consistent with those of previous studies and suggest that although HRL and CMD may contribute to the development of aggressive BC subtypes in non-Hispanic Black women, they are not directly associated with mortality disparities. These results mirror those observed for neighborhood deprivation, whereby White women living in neighborhoods defined as deprived are more likely to experience poor outcomes than their Black counterparts.^14,35^ The collective data suggest that the structural and social determinants of health faced by Black women are robust and interacting; the isolation of one domain (neighborhood in this instance) does not mitigate insults from others—that is, a piling of effects. In contrast, White women experienced fewer structural and social determinants of health, hence we observed more robust effects when considered in isolation. Our observations reinforce the concept of race as a social construct and emphasize the need for a more nuanced investigation of the differing social and structural drivers of disparate BC outcomes. Additional methods and approaches are needed to capture the intersecting influences from multiple social and structural domains on race disparities in BC mortality.

## Notes

Supplement 1. **eMethods.** Exposure Assessment for Historical Redlining and Contemporary Mortgage Discrimination **eFigure 1.** The Distribution of Historical Redlining (HRL), Contemporary Mortgage Discrimination (CMD), and Persistent Mortgage Discrimination (PMD), 2010 to 2017 **eFigure 2.** Directed Acyclic Graphs for Historical Redlining and BC Subtype, Stage, and Mortality **eFigure 3.** Age-Adjusted Cumulative Incidence Function Graphs **eTable 1.** Odds for Being Diagnosed With TNBC Compared With ER- and/or *EBBR2*-Positive Breast Cancer According to Census Tract Redlining Score **eTable 2.** Odds for ER-Negative Breast Cancer Compared With ER-Positive Breast Cancer According to Census Tract Historical Redlining and Contemporary Mortgage Discrimination Indices
eReferences

Supplement 2.
Data Sharing Statement
