# Supplementary material for: Historical Redlining, Persistent Mortgage Discrimination, and Race in Breast Cancer Outcomes
Source: JAMA Netw Open. 2024 Feb 20;7(2):e2356879. doi: 10.1001/jamanetworkopen.2023.56879 (PMC10879950; doi:10.1001/jamanetworkopen.2023.56879)
Supplement: Supplement 1. — eMethods. Exposure Assessment for Historical Redlining and Contemporary Mortgage Discrimination eFigure 1. The Distribution of Historical Redlining (HRL), Contemporary Mortgage Discrimination (CMD), and Persistent Mortgage Discrimination (PMD), 2010 to 2017 eFigure 2. Directed Acyclic Graphs for Historical Redlining and BC Subtype, Stage, and Mortality eFigure 3. Age-Adjusted Cumulative Incidence Function Graphs eTable 1. Odds for Being Diagnosed With TNBC Compared With ER- and/or EBBR2-Positive Breast Cancer According to Census Tract Redlining Score eTable 2. Odds for ER-Negative Breast Cancer Compared With ER-Positive Breast Cancer According to Census Tract Historical Redlining and Contemporary Mortgage Discrimination Indices eReferences [file jamanetwopen-e2356879-s001.pdf]

Supplementary Online Content

Miller-Kleinhenz JM, Barber LE, Maliniak ML, et al. Role of historical redlining, persistent mortgage discrimination, and race in breast cancer outcomes. *JAMA Netw Open*. 2024;7(2):e2356879. doi:10.1001/jamanetworkopen.2023.56879

eMethods. Exposure Assessment for Historical Redlining and Contemporary Mortgage Discrimination

eFigure 1. The Distribution of Historical Redlining (HRL), Contemporary Mortgage Discrimination (CMD), and Persistent Mortgage Discrimination (PMD), 2010 to 2017

eFigure 2. Directed Acyclic Graphs for Historical Redlining and BC Subtype, Stage, and Mortality

eFigure 3. Age-Adjusted Cumulative Incidence Function Graphs

eTable 1. Odds for Being Diagnosed With TNBC Compared With ER- and/or *ERBB2*-Positive Breast Cancer According to Census Tract Redlining Score

eTable 2. Odds for ER-Negative Breast Cancer Compared With ER-Positive Breast Cancer According to Census Tract Historical Redlining and Contemporary Mortgage Discrimination Indices

eReferences

This supplementary material has been provided by the authors to give readers additional information about their work.

© 2024 Miller-Kleinhenz JM et al. *JAMA Network Open*.

eMethods. Exposure Assessment for Historical Redlining and Contemporary Mortgage Discrimination

Historical Redlining:

An Historical Redlining (HRL) score was calculated for each census tract based on the proportion of HOLC grades contained within the census tract's geographic boundaries. The score is calculated by first converting the HOLC grades into a numeric value (A=1, B=2, C=3, D=4) and then using the land area proportion of each grade as a weight.[1-3] The result is a continuous score between 1 and 4, with lower scores indicative of better grades (a greater proportion of A and B grades) and higher scores indicative of worse grades (a greater proportion of C and D grades).

Contemporary Mortgage Discrimination:

Data were abstracted from the national database established as part of the Housing Mortgage Disclosure Act (HDMA) for the years 2010–2014.[4,5] Census tracts were each assigned a CMD score, which was estimated as the odds of denial of a mortgage application for a residence inside the census tract compared to those outside of the census tract. We calculated CMD scores separately for each metropolitan area. The index centers around a value of one, which corresponds to a census tract that received the same rate of mortgage approvals compared to other census tracts in the same metro-area.

© 2024 Miller-Kleinhenz JM et al. *JAMA Network Open*.

eFigure 1. The Distribution of Historical Redlining (HRL), Contemporary Mortgage Discrimination (CMD), and Persistent Mortgage Discrimination (PMD), 2010 to 2017

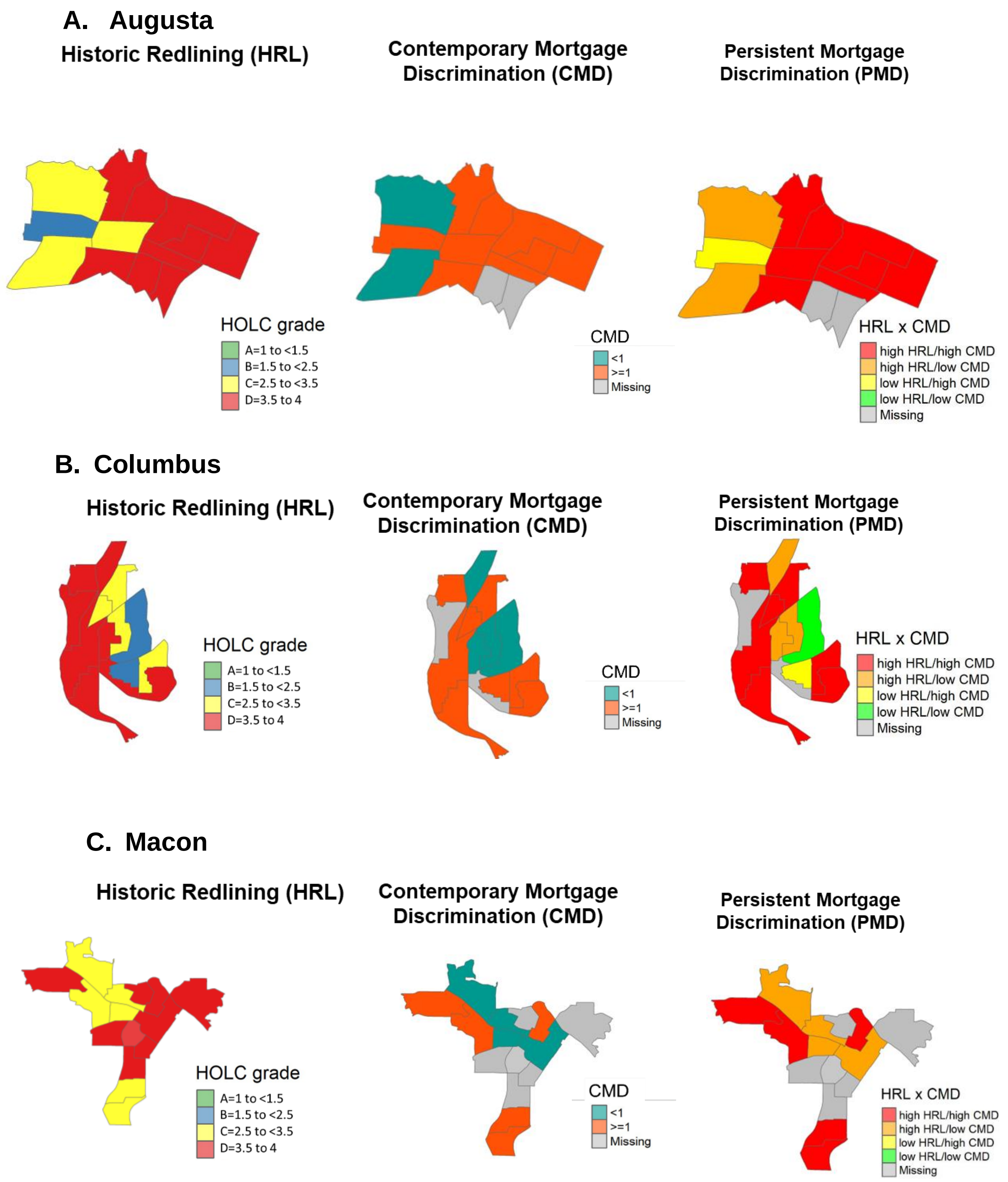

© 2024 Miller-Kleinhenz JM et al. *JAMA Network Open*.

D. Savannah

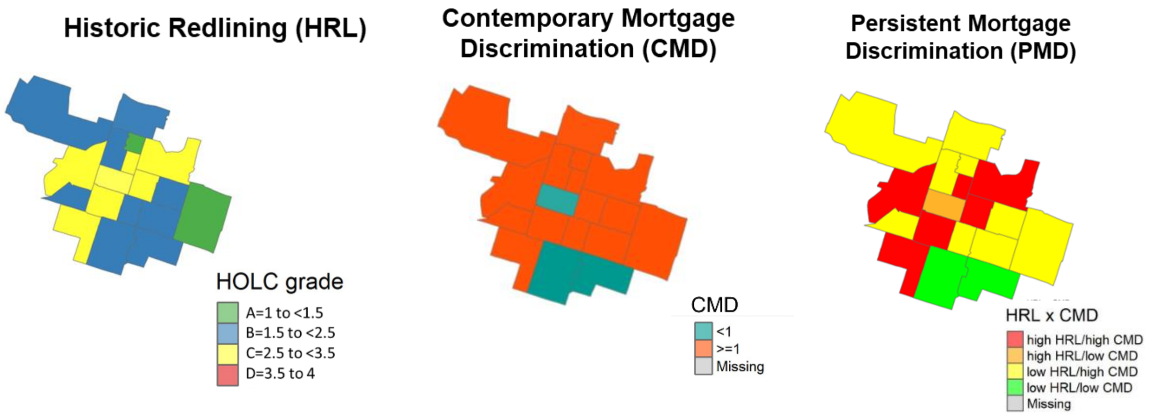

© 2024 Miller-Kleinhenz JM et al. *JAMA Network Open*.

eFigure 2. Directed Acyclic Graphs for Historical Redlining and BC Subtype, Stage, and Mortality

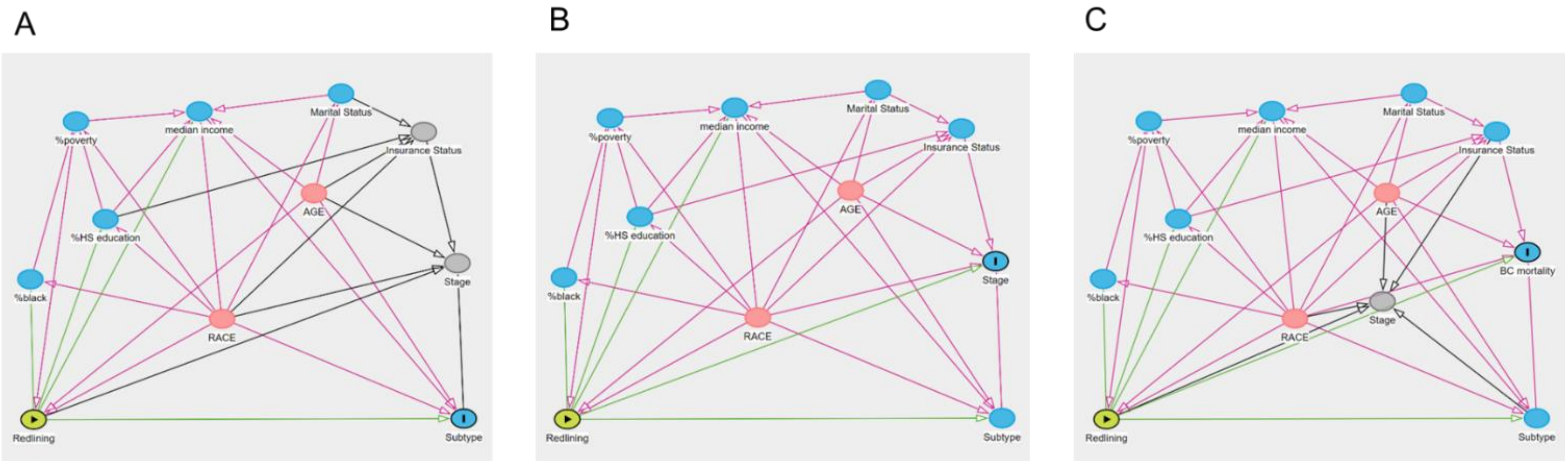

© 2024 Miller-Kleinhenz JM et al. *JAMA Network Open*.

eFigure 3. Age-Adjusted Cumulative Incidence Function Graphs

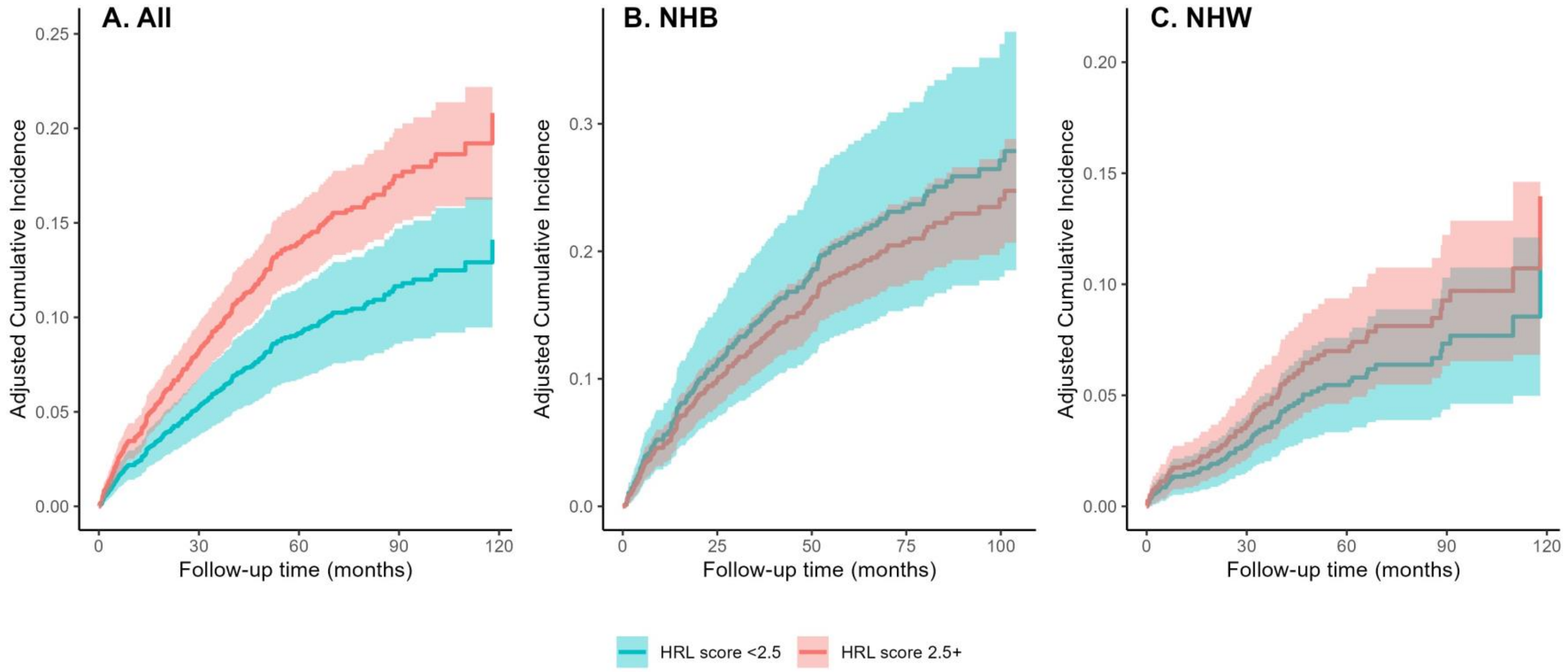

© 2024 Miller-Kleinhenz JM et al. *JAMA Network Open*.

eTable 1. Odds for Being Diagnosed With TNBC Compared With ER- and/or *ERBB2*-Positive Breast Cancer According to Census Tract Redlining Score

	ER+ and/or *ERBB2*+	TNBC	
**Historical Redlining Score**	**N**	**N**	**Age-adjusted OR (95% CI)**
**Overall**			
Continuous	1459	305	1.25 (1.07, 1.46)
Dichotomous			
<2.5	467	71	Referent
≥2.5	992	234	1.53 (1.15, 2.04)
**Non-Hispanic Black**			
Continuous	662	164	1.11 (0.87, 1.41)
Dichotomous			
<2.5	97	22	Referent
≥2.5	565	172	1.32 (0.80, 2.16)
**Non-Hispanic White**			
Continuous			0.91 (0.71, 1.18)
Dichotomous			
<2.5	370	49	Referent
≥2.5	427	62	1.08 (0.72, 1.62)

OR, Odds Ratio; CI, Confidence Interval

Data among non-Hispanic White and non-Hispanic Black women diagnosed with breast cancer in Georgia living in historically redlined areas between 2010–2017 and registered with the Georgia Cancer Registry.

© 2024 Miller-Kleinhenz JM et al. *JAMA Network Open*.

eTable 2. Odds for ER-Negative Breast Cancer Compared With ER-Positive Breast Cancer According to Census Tract Historical Redlining and Contemporary Mortgage Discrimination Indices

Race x Mortgage Discrimination (Historic and Contemporary)	ER +	ER -	Age-adjusted HR (95% CI)	MV-adjusted HR (95% CI)[a]	Early Stage	Late Stage	Age-adjusted OR (95% CI)	MV-adjusted OR (95% CI)[a]
NHW & HRL low/ CMD low	260	35	Referent	Referent	280	15	Referent	Referent
NHW & HRL high/CMD low	307	35	0.82 (0.50, 1.34)	0.81 (0.49, 1.34)	322	20	1.16 (0.58, 2.32)	1.18 (0.59, 2.35)
NHW & HRL low/CMD high	108	16	1.18 (0.63, 2.23)	1.18 (0.62, 2.23)	117	7	1.11 (0.63, 2.79)	1.10 (0.43, 2.76)
NHW & HRL high/CMD high	124	20	1.19 (0.66, 2.14)	1.11 (0.61, 2.01)	120	24	3.74 (1.90, 7.38)	3.71 (1.88, 6.87)
NHB & HRL high/CMD low[b]	138	45	2.47 (1.51, 4.03)	2.38 (1.42, 3.79)	153	30	3.65 (1.91, 7.00)	3.40 (1.77, 6.54)
NHB & HRL low/CMD high	83	21	1.96 (1.08, 3.57)	1.77 (0.97, 3.24)	81	23	5.28 (2.63, 10.6)	5.04 (2.51, 10.1)
NHB & HRL high/CMD high	323	143	3.30 (2.20, 4.95)	3.10 (2.03, 4.61)	378	87	4.30 (2.43, 7.59)	3.87 (1.22, 6.87)

HRL, Historic Redlining; CMD, Contemporary Mortgage Discrimination; HR, Hazard Ratio; CI, Confidence Interval; ER, Estrogen Receptor; MV, Multivariable [a]Age, Stage, and ER-status-adjusted [b]NHB HRL low/CRL low is excluded due to <5 patients

Data among non-Hispanic White (NHW) and non-Hispanic Black (NHB) women diagnosed with breast cancer in Georgia, 2010 to 2017, and registered with the Georgia Cancer Registry.

© 2024 Miller-Kleinhenz JM et al. *JAMA Network Open*.

eReferences

1. Lynch EE, Malcoe LH, Laurent SE, Richardson J, Mitchell BC, Meier HCS. The legacy of structural racism: Associations between historic redlining, current mortgage lending, and health. *SSM Popul Health*. Jun 2021;14:100793. doi:10.1016/j.ssmph.2021.100793

2. Meier H, Mitchell B. Historic Redlining Scores for 2010 and 2020 US Census Tracts. Ann Arbor. 2021-10-15. Ann Arbor, MI: Inter-university Consortium for Political and Social Research doi:https://doi.org/10.3886/E141121V2

3. Meier HM, B. Richardson, J. Lynch, E. Edlebi, J. . The Lasting Impact of Historic ‘Redlining’ on Neighborhood Health: Higher Prevalence of COVID-19 Risk Factors. National Community Reinvestment Coalition https://ncrc.org/holc-health/

4. 101 The 2014 Home Mortgage Disclosure Act Data (2015).

5. 77 Home Mortgage Disclosure Act: Expanded Data on Residential Lending (Federal Reserve Bulletin) 859-881 (1991).

© 2024 Miller-Kleinhenz JM et al. *JAMA Network Open*.
