# Supplementary material for: Historical Redlining, Persistent Mortgage Discrimination, and Race in Breast Cancer Outcomes
Source: JAMA Netw Open. 2024 Feb 20;7(2):e2356879. doi: 10.1001/jamanetworkopen.2023.56879 (PMC10879950; doi:10.1001/jamanetworkopen.2023.56879)
Supplement: Supplement 2. — Data Sharing Statement [file jamanetwopen-e2356879-s002.pdf]

Data Sharing Statement

Miller-Kleinhenz. Role of Historical Redlining, Persistent Mortgage Discrimination, and Race in Breast Cancer Outcomes. *JAMA Netw Open*. Published February 20, 2024.
doi:10.1001/jamanetworkopen.2023.56879

Data
Data available: No

Additional Information
Explanation for why data not available: The datasets generated during and/or analyzed during the current study are not publicly available due to IRB protocol but are available from the study PI (lauren.mccullough@emory.edu) on reasonable request.
